# Supplementary figures and images for: Type I interferon signaling induces a delayed antiproliferative response in Calu-3 cells during SARS-CoV-2 infection
Source: bioRxiv. 2023 Mar 1:2023.02.28.530557. Preprint. [Version 1] doi: 10.1101/2023.02.28.530557 (PMC10002732; doi:10.1101/2023.02.28.530557)

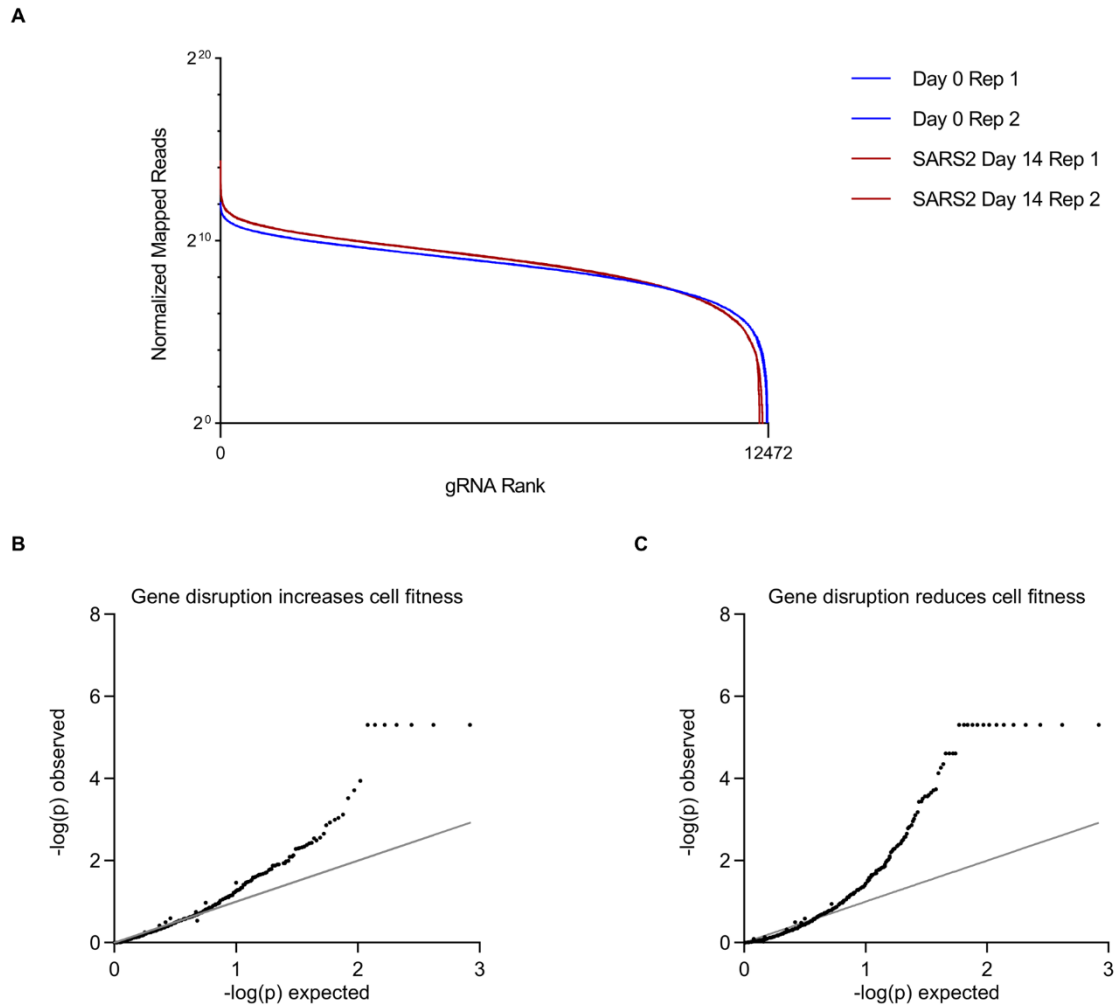

**Figure S1**

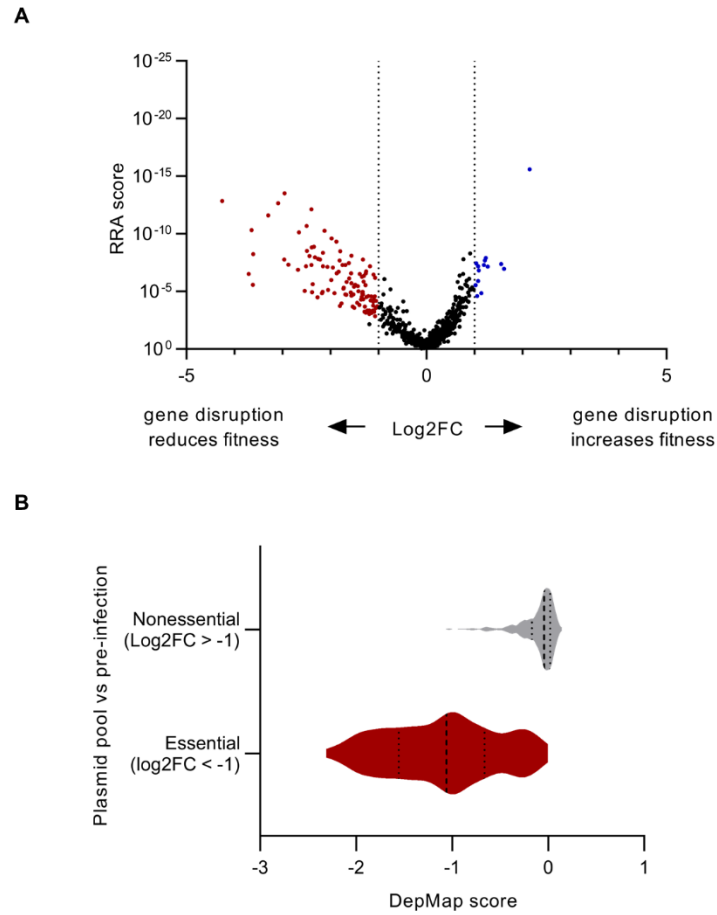

**Figure S2**

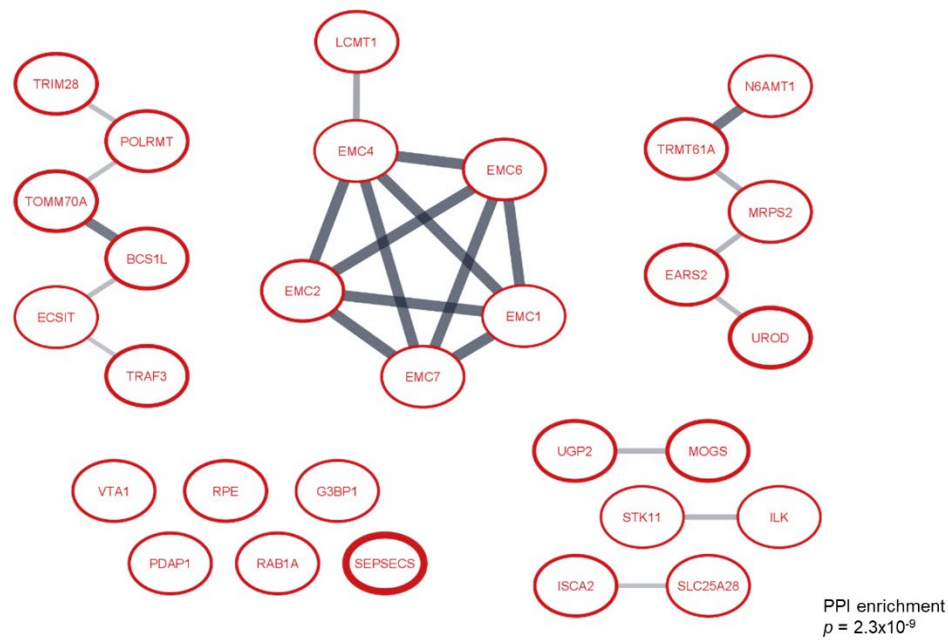

**Figure S3**

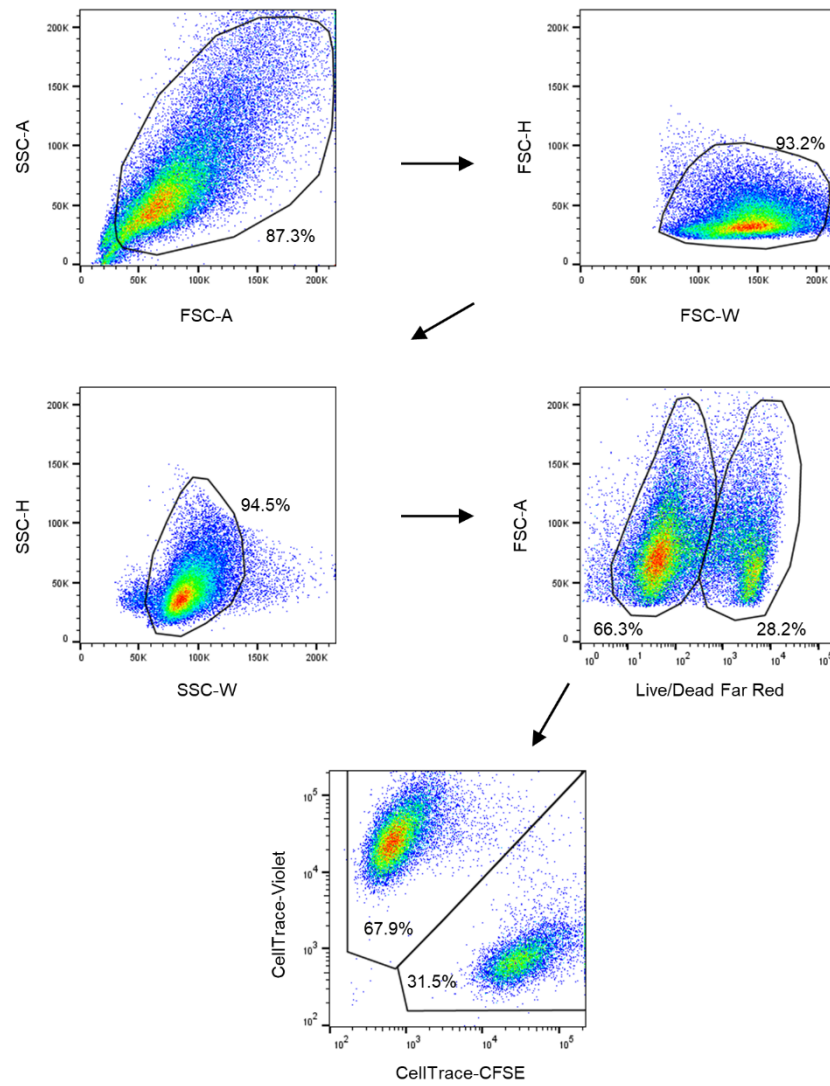

**Figure S4**

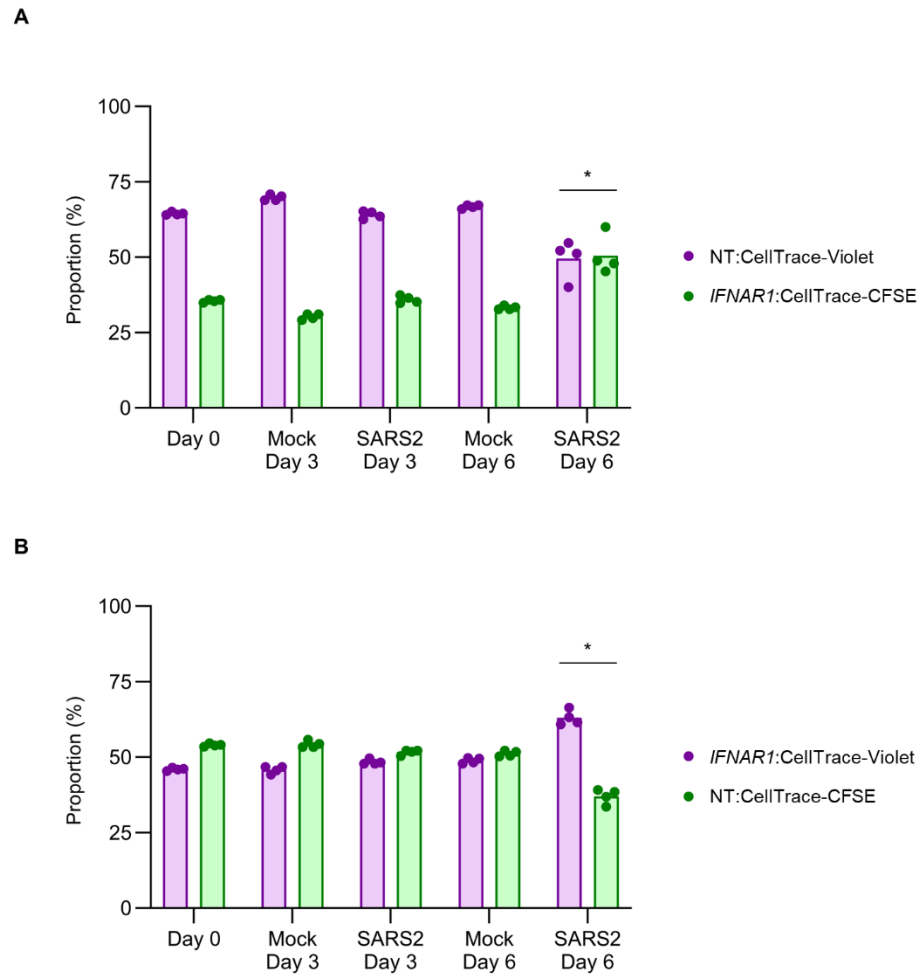

**Figure S5**

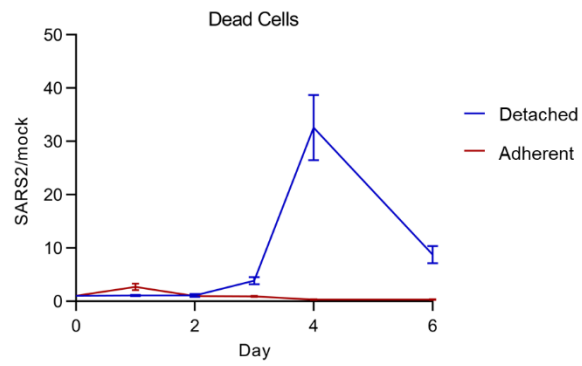

**Figure S6**

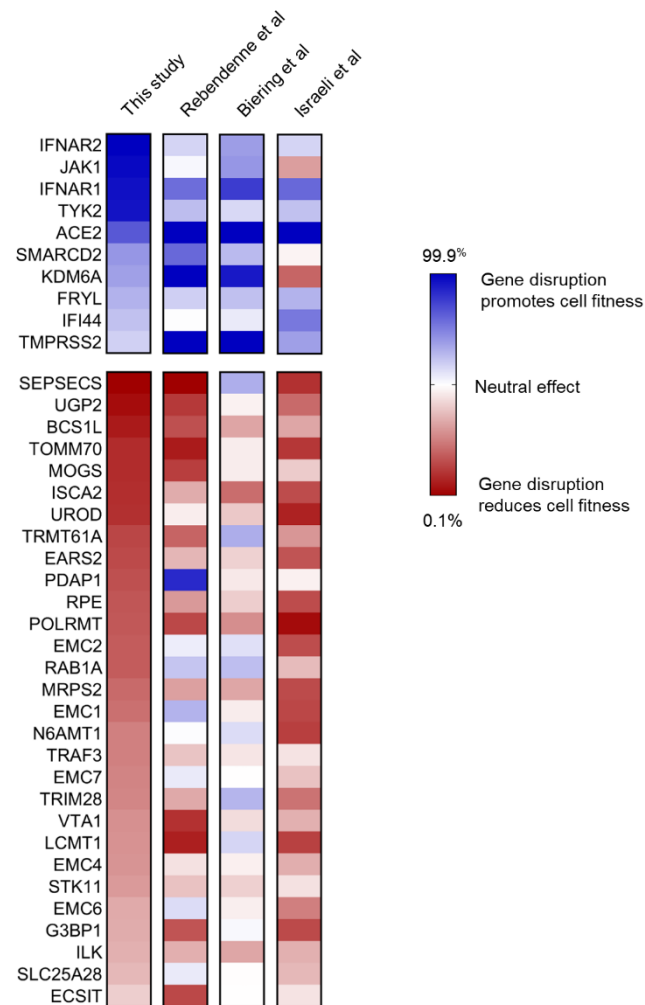

Figure S7

Supplement: Supplement 1 — Figure S1. CRISPR screen analysis. (A) Cumulative distribution functions of normalized read counts for each gRNA before and after 14 days of SARS-CoV-2 infection for each of 2 independent biologic replicates. (B) Q-Q plots of observed versus expected –log(p) for gene perturbations associated with increased (B) or decreased (C) cell fitness during SARS-CoV-2 infection for every gene targeted by the CRISPR library. Observed p-values were calculated by MAGeCK gene-level analysis (Supplemental Table 1). Figure S2. Filtering of genes with influence on Calu-3 cell fitness independently of SARS-Cov-2 infection. (A) Volcano plot of gRNA enrichment in day 0 pre-infected cells relative to the CRISPR library plasmid pool. Genes whose disruption conferred a significant (FDR<5%) increase (aggregate log2FC>1) or decrease (aggregate log2FC <−1) in cell fitness are highlighted in blue or red, respectively, and were filtered out of the CRISPR screen results for modifiers of cell fitness during SARS-CoV-2 infection. Source data is provided in Supplemental Tables 3 and 4. (B) Aggregate DepMap essentiality scores derived from CRISPR screens of 1070 cell lines for genes identified in this study as nonessential (L2FC>−1) or essential (L2FC<−1, FDR<5%) in Calu-3 cells prior to the onset of SARS-CoV-2 infection. DepMap scores of 0 indicate neutral effect on cell fitness while significant negative scores are consistent with a core essential function across cell lines. Figure S3. Established networks among genes whose disruption reduced the fitness of Calu-3 cells during SARS-CoV-2 infection. Borders of individual nodes are weighted by the - log(RRA score) in the screen, and lines connecting nodes are weighted by the strength of the protein-protein interaction within the STRING database. The significance of the number of protein-protein interactions relative to a randomly selected gene set was calculated by STRING. Figure S4. Representative flow cytometry plots. Gating strategy for quantifyi [file NIHPP2023.02.28.530557v1-supplement-1.pdf]
